# Effect of handedness on the occurrence of semantic N400 priming effect in 18- and 24-month-old children

**DOI:** 10.3389/fpsyg.2014.00355

**Published:** 2014-04-28

**Authors:** Jacqueline Fagard, Louah Sirri, Pia Rämä

**Affiliations:** Laboratoire Psychologie de la Perception, Université Paris Descartes - CNRS (UMR 8242)Paris, France

**Keywords:** semantic priming, ERPs, N400, handedness, vocabulary, children

## Abstract

It is frequently stated that right-handedness reflects hemispheric dominance for language. Indeed, most right-handers process phonological aspects of language with the left hemisphere (and other aspects with the right hemisphere). However, given the overwhelming majority of right-handers and of individuals showing left-hemisphere language dominance, there is a high probability to be right-handed and at the same time process phonology within the left hemisphere even if there was no causal link between both. One way to understand the link between handedness and language lateralization is to observe how they co-develop. In this study, we investigated to what extent handedness is related to the occurrence of a right-hemisphere lateralized N400 event related potential in a semantic priming task in children. The N400 component in a semantic priming task is more negative for unrelated than for related word pairs. We have shown earlier that N400 effect occurred in 24-month-olds over the right parietal-occipital recording sites, whereas no significant effect was obtained over the left hemisphere sites. In 18-month-olds, this effect was observed only in those children with higher word production ability. Since handedness has also been associated with the vocabulary size at these ages, we investigated the relationship between the N400 and handedness in 18- and 24-months as a function of their vocabulary. The results showed that right-handers had significantly higher vocabulary size and more pronounced N400 effect over the right hemisphere than non-lateralized children, but only in the 18-month-old group. We propose that the emergences of right-handedness and right-distributed N400 effect are not causally related, but that both developmental processes reflect a general tendency to recruit the hemispheres in a lateralized manner. The lack of this relationship at 24 months further suggests that there is no direct causal relation between handedness and language lateralization.

## Introduction

It is frequently stated that right-handedness reflects hemispheric dominance for language (for instance, left hemisphere for phonological processing and right hemisphere for prosody). One explanation often given is that the main language functions are processed by the left hemisphere and that the left-hemisphere is specialized for processing fast temporal transitions, which are involved both in language and in precision skills (for review, see Minagawa-Kawai et al., 2011). Yet, the basis on which it is argued that language lateralization and handedness are related is that most right-handers also process the phonological aspects of language with their left hemisphere, in typical (Knecht et al., 2000) and atypical (Frey, 2008) populations. However, even if two factors completely independent were driving 90% of the population toward right-hand preference for one, and 92% of the population toward processing phonological aspects of language in the left hemisphere for the other one, statistical calculations show that chances that an individual is right-handed and processes language with the left hemisphere would be as high as 83%. Thus, other arguments than correlations are needed to decide whether right-handedness and brain asymmetries in language processing have any cross causality or share a common causality. One argument could be that handedness and hemispheric specialization for language develop in close relation to each other, for instance that one influences the development of the other. In adults, the N400 effect in semantic priming tasks is often distributed over the right hemisphere (Bentin et al., 1985; Kutas et al., 1988; Van Petten and Luka, 2006) and as a first step toward evaluating the relation between handedness and language lateralization during development, we investigated toddlers' handedness and right-hemisphere N400 semantic priming effect during language processing.

## The development of language, of language lateralization, and of handedness

Both handedness and language lateralization have their source very early in life. Concerning handedness, a predominant use of the right hand in most fetuses has been observed as early as 15 weeks of gestational age (Hepper et al., 1991), and this is related with hand preference 12 years later (Hepper et al., 2005). When reaching becomes clearly cortically controlled, after 4–5 months of age, infants show hand preference (Michel et al., 2006), in particular when grasping requires precision (Fagard and Lockman, 2005). Infants show hand preference as soon as they start mastering a new complex skill, such as bimanual complementary actions (Potier et al., 2013) or tool use (Rat-Fischer et al., 2013). In addition, hand preference for reaching only slightly and non-significantly increases from 6 to 7 months to the second year of life (Jacquet et al., 2012). Thus, by 18 months of age handedness is rather well established, at least for the majority of infants.

As regards language lateralization for perception, very early signs have been observed. At birth, some studies using habituation (Bertoncini et al., 1989) or auditory reinforcement (DeCasper and Prescott, 2009) in non-nutritive sucking showed a right ear advantage for processing changes in syllables but this has not been always confirmed in other behavioral studies (Vargha-Khadem and Corballis, 1979; Best et al., 1982). However, a recent functional Magnetic Resonance Imaging (fMRI) study has shown more activation of the left hemisphere in processing changes in syllables in 29-week premature infants (Mahmoudzadeh et al., 2013; see also Kasprian et al., 2011). In addition, other brain imaging studies confirmed left-hemisphere greater activation for phonological processing at or around birth (Pena et al., 2003; Gervain et al., 2008). This early lateralization is compatible with earlier data on structural asymmetry of the language areas of the brain observed in post-mortem fetal (Chi et al., 1977) and *in vivo* brain imaging infant studies (Dubois et al., 2009). Concerning the functions typically involving the right hemisphere in adults, such as processing of pitch contour and prosody, it appears to be processed by the right hemisphere already at 3 months of age (Homae et al., 2006; Grossmann et al., 2010).

Lateralization of language production has also received interest: for instance, Trevarthen noted that the first cooings are often accompanied by movements of the right hand (Trevarthen, 1996). Mouth opening during babbling, but not during smiling, is asymmetrical to the right side (Holowka and Petitto, 2002). Communicative pointing, more often right-handed than object grasping (Cochet and Vauclair, 2010; Cochet et al., 2011; Esseily et al., 2011), is lateralized almost from its start (Blake et al., 1994; Vauclair and Imbault, 2009; Jacquet et al., 2012). Finally, symbolic gestures are more often performed with the right hand than non-symbolic gestures (Bates and Dick, 2002).

There are a few studies on the relation between language development itself and handedness. For instance, according to Ramsay (1984) infants begin to demonstrate unimanual right-handedness on the week of babbling onset, whereas they don't show any significant hand preference on the preceding week(s). A more recent longitudinal study has shown that when hand preference is evaluated between 6 and 14 months, the group of infants clearly categorized as right-handed was significantly more advanced in language evaluated by Bayley scales at 24 months than the group of infants categorized as having uncertain hand preference (Michel et al., 2013). It was also found that the amount of communicative pointing, a recognized prelinguistic skill (Bates et al., 1975), was related to handedness (Cochet et al., 2011; Esseily et al., 2011).

In contrast, the studies on the relation between the development of language lateralization and handedness are scarce and the few existing studies are not in favor of a strong relationship between both asymmetries during early development. For instance, in the communicative pointing longitudinal studies left-handers for grasping were often observed to be right-handed for pointing, and no correlation between developmental change in handedness for pointing and for grasping was observed (Vauclair and Imbault, 2009; Cochet and Vauclair, 2010; Jacquet et al., 2012). However, comparing hand use for communicative pointing with hand use for grasping objects is an indirect way to establish a relation between language lateralization and handedness. To our knowledge, no studies tackled the question of the relationship between the development of language lateralization and the emergence of handedness. In the study presented here we observed the relationship between handedness and the right-lateralized N400 event-related potential (ERP) in a semantic priming task.

Semantic priming provides a tool to study the organization of words in lexical-semantic memory (e.g., Meyer and Schvaneveldt, 1971; Kutas and Hillyard, 1989; Lucas, 2000). In ERP studies in adults, a negative waveform that peaks between 350 and 550 ms post-stimulus onset is more negative for unrelated than for related prime-target word pairs (e.g., Bentin et al., 1985; Holcomb, 1988; Brown et al., 2000). This is called the N400 effect. The N400 effect is typically strongest over the central and parietal recording sites, and it is stronger over the right hemisphere recording sites in adults, especially for written words (e.g., Bentin et al., 1985; Kutas et al., 1988; Van Petten and Luka, 2006), but more symmetrically distributed for auditorily presented words (for review, see Van Petten and Luka, 2006).

In our recent study, we recorded the ERPs during an auditory semantic priming task in young children in order to ascertain whether words in long-term semantic memory storage are organized by their semantic relatedness in 18- and 24-month-olds (Rämä et al., 2013). The results showed that the N400-like priming effect occurred in 24-month-olds over the **right** parietal-occipital recording sites. In 18-month-olds, the effect over the right parietal-occipital recording sites was observed similarly to 24-month-olds only in those children with higher word production ability. This is in accordance with previous studies showing that the right-lateralized N400 response is dependent on productive skills (Friedrich and Friederici, 2004, 2010; Torkildsen et al., 2006) suggesting right-hemispheric distribution might reflect maturity in lexical-semantic processing. Typically, the second year of life is associated with a significant increase in word comprehension and production (Bloom, 1973; Reznick and Goldfield, 1992; Meints et al., 1999; Ganger and Brent, 2004). This vocabulary burst is suggested to be related to advancing in word segmentation, development of naming insight, and ability to categorize objects (for review, see Ganger and Brent, 2004).

The influence of handedness on the magnitude of N400 has never been reported in children. Since right-handedness has been associated with advanced language processing in early childhood, as seen previously, we hypothesized that not only vocabulary size but also handedness would be related to the occurrence of the N400 effect. In our previous study (Rämä et al., 2013), we did not report the results of handedness evaluation but handedness was evaluated in most of the children who participated to the study. In the current study, we included only those children whose handedness, vocabulary, and N400 effect was measured, and we reanalyzed our data.

## Methods

### Subjects

Sixteen (5 girls and 11 boys) 18-month-old (range: 17 months 21 days to 19 months 2 days) and sixteen (11 girls and 5 boys) 24-month-old (range: 23 months 24 days to 25 months 24 days) children from monolingual French-speaking families were included in the current study. The parents gave informed consent before participation. The comprehensive and productive vocabulary size was tested by a French translation and adaptation of the MacArthur Communicative Development Inventory for Words and Sentences (CDI; Fenson et al., 1993). Parents filled the CDI at home, within a week or two after the experiment. Eleven additional children were rejected from original study (Rämä et al., 2013) since they did not pass the handedness test and/or parents did not provide the CDI. All children were born full-term and none of them suffered from hearing or language impairment.

### Handedness evaluation

We used the baby handedness test (BbHtest, Sacco et al., 2006). The BbHtest comprises five items to test simple grasping and two items to test precision grasping. Objects for testing *simple grasping* were small baby toys: three Playmobil® figurines, one hand-shake toy (maracas) and a teether. For *precision grasping*, one task consisted in taking a very thin red tube (6 mm in diameter) inserted in a slightly shorter transparent tube from which only the top protruded and the other task consisted in grasping a small horse inserted in a container that was 30 mm in height. To favor unimanual grasping, these two objects were presented so that the infants could not grasp the container, but only the object inside. The baby laterality test thus comprised seven items in total. All objects were presented within reaching distance of the infant at a midline position.

### Word stimuli

The stimuli were one-, two-, or three-syllable French basic level nouns from seven different categories (animals, clothes, body parts, food, furniture, transportation, and household items). The word categories were chosen from the CDI. The stimuli were arranged into 72 prime-target word pairs (see, for details Rämä et al., 2013). There were 36 words for each trial type (unrelated primes, related primes, and target words). Half of the word pairs consisted of categorically (but not associatively) related words (e.g., train-bike) and half of them of categorically unrelated words (e.g., chicken-bike). Each target word was presented twice; once in the related and once in the unrelated condition. The same word pairs were presented twice during the experiment. The words were recorded and edited with Cool Edit 2000 (Syntrillium Software Corp., Phoenix, AZ) and Pratt (version 5.3.02) programs. The sound levels were normalized among the speakers and words. The speakers were four native French female speakers and they were asked to pronounce the words slowly. Prime and target words in a given trial were always spoken by a different speaker not to allow children to rely on acoustic features. In addition, it had been shown that the speaker variability facilitates word learning in children (Richtsmeier et al., 2009).

### Experimental procedure

During the EEG recordings, children were seated on their caregiver's lap or by themselves in a dimly lit room facing loudspeakers and a computer screen at the distance of 100–120 cm. Parents were informed of the purpose of the study before signing the consent. They were instructed not to communicate verbally or non-verbally with their child during the actual experiment. To keep the children distracted during the experiment, they were allowed to play with small toys positioned on the table in front of them during the experiment. Also colorful pictures from children's books were presented on the computer screen during the experiment but they were not synchronized with auditory stimulation. Children were allowed to choose to look at the pictures or play with the toys. There was no relatedness between words and pictures. A new picture appeared every 15 s.

The interstimulus interval (ISI) was 200 ms between the prime and the target words in each word pair and the intertrial interval (ITI) between the word pairs was 2200 ms. Stimulus onset asynchrony (SOA) varied between 635 ms and 1266 ms (mean SOA = 910 ms, *SD* = 166 ms). The experiment was divided into four blocks, and there were short breaks between the blocks. Words from different semantic categories were randomly distributed across the blocks. Each word pair was repeated twice during the experiment, but never within the same block. The handedness evaluation was performed either before or after the EEG experiment. The whole experiment lasted 10 min. The study was approved by the Ethical Committee of the University of Paris Descartes, and the experimental procedure was conducted in accordance with the principles of the Declaration of Helsinki (1964).

### EEG recordings

Continuous electroencephalogram (EEG) was recorded (bandpass = 0.1–100 Hz, sampling rate = 250 Hz) from 62 electrodes using a Geodesic Sensor Net (GSN, NetStation EGIS V2.0, with 10–10 international electrode system) referenced to the vertex during the acquisition. Impedances were kept below 50 kΩ. EEG was filtered (0.3–30 Hz), segmented (1200 ms, beginning 200 ms before target word onset to 1000 ms post-stimulus), and ocular artefacts were removed with an ocular artefact removal (OAR) algorithm (Gratton et al., 1983). The 200-ms pre-stimulus period determined the baseline for amplitude measures. The epochs including artefacts (eye-movements, blinks, motion artefacts exceeding ± 150 μV in any channel) were automatically excluded. Epochs including more than 20 contaminated channels were rejected as well. Channels marked as bad were replaced with other channels in proximity using spherical spline interpolation. The epochs were averaged separately for each subject and type of target (related and unrelated) word. The averaged waveforms were re-referenced to the average reference and baseline corrected. The epochs were grand-averaged across all participants in each age group for the type of target word. In the original study, participants with less than 10 trials *per* target word type were rejected. The mean number of trials after the artefact rejection was 26 (13–42 trials) and 22 (11–51 trials) for related and 25 (13–39 trials) and 20 (10–50 trials) for unrelated target words in 18- and 24-month-olds, respectively.

### Data analyses

#### Handedness

To assess handedness on the BbHtest, a laterality index (LI) was calculated using a classical formula [RH grasps − LH grasps/(RH grasps + Lh grasps + bimanual grasps)] (Michel et al., 2002; Fagard and Lemoine, 2006). From the LI, the children were characterized as right-handers (LI ≥ 0.5), left-handers (LI ≤ −0.5), or non-lateralized (LI comprised between −0.51 and 0.49).

#### Vocabulary

The participants in each age group were divided into two vocabulary groups based on their productive vocabulary scores obtained in McArthur Communicative Development Inventory for Words and Sentences. The mean vocabulary score was calculated for each participant and the median score of all participants was used to divide them into two groups, named low and high producer groups. The mean number of words produced by 18-month-olds was 43 (*SD* = 54, median = 24.5). Here we decided to eliminate, for the analyses as a function of the vocabulary, two 18-month-old children whose number of words was too close to the median (24 and 25 words). The mean number of words produced by 24-month-olds was 241 (*SD* = 154, median = 269.5). We also eliminated, for the analyses as a function of vocabulary group, one 24-month-old child whose number of words was close to the median (261 words), and lower than the median but higher than the mean.

#### ERPs

In the original study (Rämä et al., 2013), a significant N400 effect was obtained over the right posterior-parietal recording sites. The magnitude of N400 component in response to related and unrelated target words was measured by calculating the mean amplitude of the component within 200-ms-windows. To analyze the significance of the component, a repeated measure of analysis of variance (ANOVA) included as within subject factors: trial type (related vs. unrelated), area (frontal, central, and parietal-occipital), hemisphere (left vs. right), and time interval (five 200-ms time windows starting from 0 to 1000 ms), and as a between subject factor the vocabulary (high producers vs. low producers). The data were analyzed using the SPSS statistical package (IBM SPSS statistics, version 20) and all ANOVA results were Greenhouse-Geisser corrected. According to the 10–10 international electrode position system, the sensor positions of the right parietal-occipital area were the following: P2, P6, P8, P10, PO4, PO8, O2, and TP10. The N400 effect was more pronounced for unrelated than for related targets during the first, second, and the third time intervals over the right hemisphere [*t*_(22)_ = 2.34–3.23, *p* < 0.05–0.005]. Here, we report the results of the effect of handedness and vocabulary on the magnitude of this previously found significant right-lateralized N400 effect.

#### Statistical analyses

Chi-square tests were used to analyze the distribution of right-handed vs. non-lateralized children as a function of vocabulary. We used ANOVA to test the effect of age, level of vocabulary and handedness on the N400. Finally, we calculated correlations between the raw values of LI, number of words and N400.

## Results

### Vocabulary

At 18 months, in the low producer group, the average score was 8 words (*SD* = 4.5; range: 0–15 words) and in the high producer group the average score was 83.3 words (*SD* = 62.7; range: 29–214 words). At 24 months, in the low producer group, the average score was 102 words (*SD* = 91.3; range: 4–243 words) while the average score in the high producer group was 360 words (*SD* = 90.1; range: 278–555 words).

### Handedness

The LI increased slightly but not significantly (*p* = 0.20) between 18- (*m* = 0.36, *SD* = 0.5) and 24-month-olds (*m* = 0.58, *SD* = 0.5). There were more right-handed than non-lateralized children and only one left-hander in each age group (see Table 1). A chi-square on the distribution of handedness as a function of age showed also no significant age effect (*p* = 0.84).

**Table 1:** **Distribution of handedness category based on the laterality index in 18- and 24-month-olds**.

	**18-months**	**24-months**	**All subjects**
Right-handers	9 (56.2%)	11 (68.7%)	20 (62.5%)
Non-lateralized	6 (37.2%)	4 (25%)	10 (31.2%)
Left-handers	1 (6.2%)	1 (6.2%)	2 (6.2%)
Total	16	16	32

### Relationships between vocabulary, N400 effect, and handedness

#### Vocabulary and handedness

Since there were only two left-handers, we did not include them in any statistical analysis, but they are briefly mentioned and their values are indicated on the graphs.

**Figure 1:**
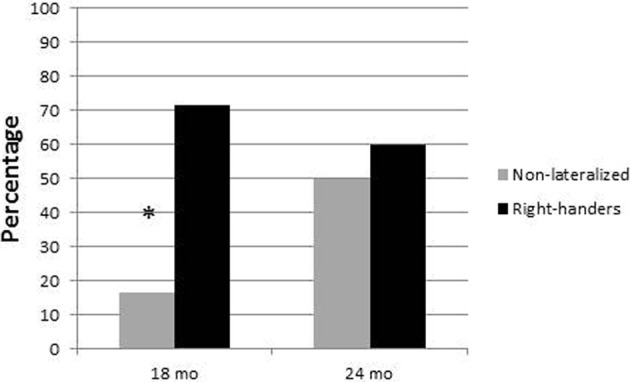
**Percentage of children with high vocabulary as a function of age and handedness.** ^*^p <0.05.

At 18 months, the proportion of children with a high vocabulary score was greater among right-handers (71.4%) than among non-lateralized (16.7%, see Figure 1). A chi-square on the distribution of handedness as a function of vocabulary at 18 months showed a significant effect [χ^2^_(1)_ = 3.9, *p* < 0.05]. At 24-months, the proportion of children with a high vocabulary score was only slightly greater among right-handers (60%) than among non-lateralized (50%), and a chi-square on the distribution of handedness as a function of vocabulary at 24 months showed no significant effect (*p* = 0.73). The correlations between number of words and LI were 0.38 at 18 months and 0.06 at 24 months.

#### N400 effect and handedness

**Figure 2:**
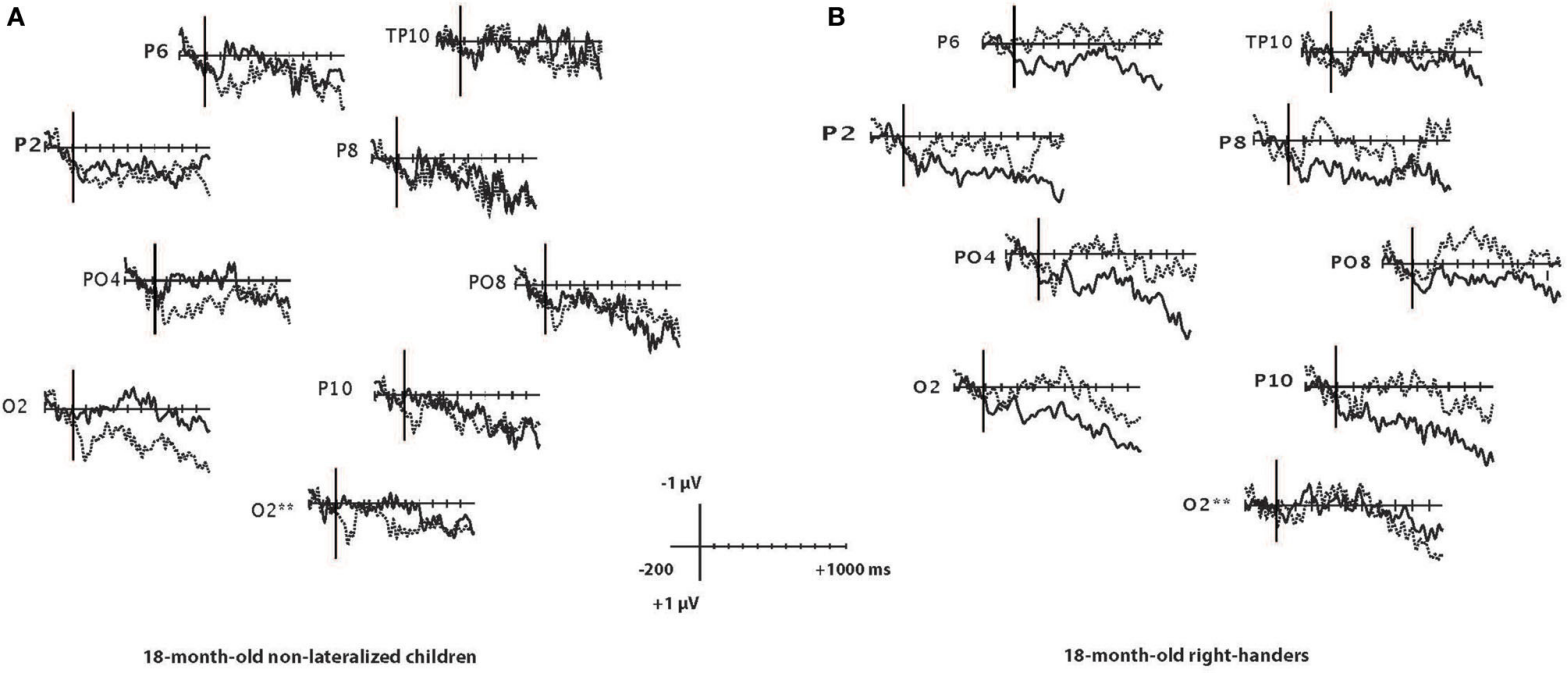
**(A,B)** Grand-averaged waveforms for related (solid line) and unrelated (dashed line) target words in 18-month-old right handers **(A)** and non-lateralized **(B)** children over the right parietal-occipital recording sites. According to the 10–10 international system of electrode positions, channels 40 and 44 are both indicated as O2. The O2^**^ reflects channel 44. The vertical line illustrates the target word onset.

**Figure 3:**
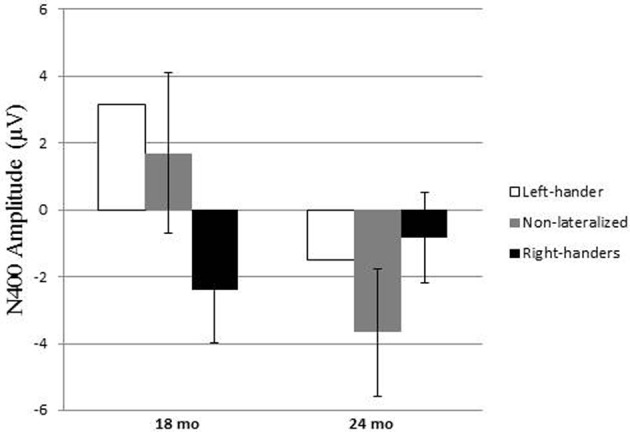
**The N400 effect size as a function of age and handedness (The two left-handers are represented here even though, for obvious reason, they were not included in the ANOVA)**.

At 18 months, only the right-handers had a right-distributed N400 effect whereas 24-month-olds from all handedness categories had the N400 effect (see Figures 2, 3). An ANOVA of the N400 as a function of age and category of handedness (non-lateralized vs. right-handed) showed no significant main effects of age (*p* = 0.18) or category of handedness (*p* = 0.63), but the interaction between age and category of handedness was significant [*F*_(1, 26)_ = 6.3, *p* < 0.02]. A Fisher LSD *post-hoc* test indicated that the N400 effect obtained in non-lateralized children differed significantly from that in right-handed children at the age of 18-months (*p* < 0.05), but not at 24 months (*p* = 0.18). The correlations between N400 and LI were −0.52 at 18 months (*p* < 0.05) and 0.29 at 24 months.

#### N400 effect, vocabulary, and handedness

Finally, we looked at the N400 as a function of age, handedness and vocabulary. As can be seen in Table 2, at 18 months the right-handers with high vocabulary had the most negative N400 effect and the non-lateralized children with low vocabulary was the only group without N400. At 24 months, the non-lateralized children with a high vocabulary had the most negative N400. Children with high vocabulary (right-handed and non-lateralized) had a slightly larger N400 than children with low vocabulary. An ANOVA was calculated on the N400 effect with age (x 2), handedness (x 2, Right-handed vs. Non-Lateralized) and vocabulary (x 2, High vs. Low) as independent variables. It showed no main effect of age (*p* = 0.57), no main effect of handedness (*p* = 0.97) but a main effect of vocabulary [*F*_(1, 19)_ = 5.7, *p* < 0.05]. None of the interactions were significant. A *post-hoc* LSD test showed that, within the same age groups, the 18-month-old right-handers with high vocabulary and the 18-month-old non-lateralized children with low vocabulary differed significantly (*p* < 0.01); similarly, the 24-month-old non-lateralized children with high vocabulary differed significantly from the right-handers with low vocabulary (*p* < 0.05).

**Table 2:** **N400 effect (in μV) over the right hemisphere as a function of vocabulary and handedness**.

	**18-months low voc.**	**18-months high voc.**	**24-months low voc.**	**24-months high voc.**
Right-handers	−1.13 (0.9, *N* = 2)	−3.9 (3.5, *N* = 5)	0.48 (3.7, *N* = 4)	−1.4 (1.9, *N* = 6)
Non-lateralized	2.3 (5.3, *N* = 5)	−1.39 (*N* = 1)	−0.4 (1.6, *N* = 2)	−6.7 (0.06, *N* = 2)

## Discussion

The goal of this study was to investigate whether handedness and the occurrence of right-distributed N400 effect in a semantic priming task are related in 18- and 24-month-old children of low vs. high level of vocabulary. Our results showed *a significant relationship between handedness and level of vocabulary* in 18-month-olds. At that age, the proportion of children with a high vocabulary was greater among right-handers than among non-lateralized children. This is in line with evidence obtained in a recent study showing that children who showed consistent right-handedness between 6 and 14 months of age had more vocabulary at the age of 24 months than children whose handedness was expressed later (Nelson et al., 2014).

In our study, at 24 months, the non-lateralized children did not differ significantly from the right-handers for vocabulary. This may indicate that being right-handed (or having a preferred hand, more left-handers should be tested) early in life may be associated with a more precocious development of vocabulary, but that right-handedness *per se* has not a lasting influence on the level of vocabulary.

The greater percentage of right- than left-handers in our sample and also its slight (but non-significant) increase with age is in accordance with previous findings (Cochet et al., 2011; Jacquet et al., 2012). It has been found that handedness is already evident at 18 months, even though the percentage of non-lateralized participants at that age is higher than that of adults (Fagard, 2013) and even though there are large fluctuations in infants hand preference (Fagard, 1998; Corbetta and Thelen, 2002).

We also found *a relationship between the right-hemisphere distributed N400 effect and handedness* in 18-month-olds. The occurrence of the N400-like response in children has earlier been associated with incongruence detection in a picture-word context (e.g., Friedrich and Friederici, 2004; Torkildsen et al., 2006) and with semantic priming (Torkildsen et al., 2007; Rämä et al., 2013). It has been shown that there is a strong relationship between early word acquisition and generation of N400 response in developing brain (Friedrich and Friederici, 2010). Recently, the N400 effect was found even in 6-month-olds after few exposures of novel object-word combinations, suggesting that the mechanisms of N400 are mature already very early in infancy (Friedrich and Friederici, 2011). In the current study, the right-handed 18-month-olds had significantly more pronounced N400 effect than the non-lateralized 18-month-olds. The influence of handedness and vocabulary size on the amplitude of the N400 effect in 18-month-olds may be confounded since there is a link between them. Disentangling them was limited by the fact that there was only one 18-month-old who, at the same time, was non-lateralized and had a high vocabulary. However, the *post-hoc* comparisons of the N400 effect in 18-month-old right-handed children with either a low or a high vocabulary showed that the difference was not significant (*p* = 0.34), and the same was observed when comparing 18-month-old non-lateralized children with either a low or a high vocabulary (*p* = 0.33). This means that level of vocabulary alone cannot account for the larger amplitude of the N400 effect in 18-month-old right-handers. Similarly, the *post-hoc* comparisons of the N400 effect in 18-month-olds with a low vocabulary showed that the difference between right-handed and non-lateralized children was not significant (*p* = 0.24), and the same was observed when comparing 18-month-olds with a high vocabulary as a function of handedness (*p* = 0.50). This means that handedness alone cannot account for the variation of amplitude of the N400 effect. The group exhibiting the largest N400 effect included children who were right-handed and had a high level of vocabulary and the group who lacked the N400 effect included children who were not lateralized and had a low level of vocabulary. Thus, the relation between handedness and right-hemisphere N400 effect at 18 months seems to be partly, but not completely, mediated by the level of vocabulary.

At 24 months, there was no significant difference in the amplitude of the N400 effect between right-handed and non-lateralized children when vocabulary was not considered. No main effect of vocabulary had been observed in the previous study at that age (Rämä et al., 2013). Here we show that the N400 effect was significantly larger in the non-lateralized children with high vocabulary than in the right-handers with low vocabulary. Thus, at 24 months, there was no association between right-handedness and right-hemisphere N400 semantic priming effect, but vocabulary skills may still influence right-hemisphere N400 semantic priming effect in non-lateralized children. More data would be needed to confirm this.

The relation between the right-lateralized N400 effect and the level of vocabulary has been previously shown, even in 12-month-olds, as mentioned in the introduction (Friedrich and Friederici, 2004, 2010; Torkildsen et al., 2006). All these results, including ours, suggest that infants, as long as they have developed a certain level of productive vocabulary skills, demonstrate a similar asymmetrical N400 distribution than older children and adults (Bentin et al., 1985; Kutas et al., 1988; Van Petten and Luka, 2006; however, see Kutas and Hillyard, 1980, or Ressel et al., 2008; Spironelli and Angrilli, 2009, for different results concerning the asymmetry of N400 in adults). In all these infant studies of the N400 effect, handedness was never reported.

To our knowledge, this is the first ERP study to report a transitory relation between the N400 priming effect, vocabulary skills, and handedness in 18-month-old children. This period of age is characterized by the vocabulary “spurt,” known to occur during the second year of life when an important increase in word production is observed (e.g., Bloom, 1973; Reznick and Goldfield, 1992). Our results indicate that both handedness and vocabulary skills contribute to the occurrence of the N400 effect during a semantic priming task at 18 months, showing for the first time a link between handedness and language lateralization in infants.

How can we interpret the link between handedness and language lateralization? Since our results, like the previous ones already mentioned, support the notion that language is lateralized from its start, the same hypotheses that were evoked for the link between handedness and language development could in theory be applied here. The link between handedness and language development has been interpreted as reflecting the reorganization of hemispheric specialization (Ramsay, 1984), and as expressing the role of the left hemisphere in both language and right-handedness (Nelson et al., 2014). Does it mean that handedness is favored by lateralized language development or, alternately, that lateralized language development is triggered by the emergence of handedness? Here we cannot make the hypothesis that 18 month-olds are right-handed because of high vocabulary skills and right-distributed N400 effect since there are signs of handedness already *in utero* (Hepper et al., 1991), and since right-handedness predicts vocabulary skills later on (Nelson et al., 2014). Alternately, some argue that right-handedness may give an advantage for creating symbolic representations which is expressed by an ability to manage simultaneously multiple objects, an ability which is more developed in consistent right-handed infants than in inconsistent-handed infants (Kotwica et al., 2008), and that may favor language development (Nelson et al., 2014). The fact that neither the level of vocabulary or right-handedness alone did guarantee a significant N400 effect at the age of 18 months in our study may indicate that both high vocabulary skills and right-handedness reflect a lateralization advantage, without one being the cause of the other. In addition, the fact that we found a right-hemisphere language function to be more developed at 18 months in right-handers than in non-lateralized children may show that a more general lateralization effect is involved rather than only left-hemisphere facilitation. This is interesting to relate to a recent study showing a link between the density of gray matter in the right hippocampus at 7 months and expressive language skills at 12 months (Can et al., 2013).

In conclusion, our results confirm a link between the development of right-handedness and vocabulary skills and show a link between right-handedness and language lateralization at 18 months. We propose that the emergence of right-handedness and of right-distributed lexical-semantic processing, rather than being causally related one way or another, both reflect a general tendency to recruit the two hemispheres in a lateralized manner. The lack of relationships at 24 months may indicate that the relation between right-handedness and language lateralization at an earlier age does not correspond to a direct causal relationship.

### Conflict of interest statement

The authors declare that the research was conducted in the absence of any commercial or financial relationships that could be construed as a potential conflict of interest.
